# Simultaneous Determination of Cortisol and Cortisone from Human Serum by Liquid Chromatography-Tandem Mass Spectrometry

**DOI:** 10.1155/2014/787483

**Published:** 2014-03-06

**Authors:** Sanghoo Lee, Hwan-Sub Lim, Hye-Jin Shin, Seol-A Kim, Jimyeong Park, Hyun-Chul Kim, Hyogyeong Kim, Hyung Joo Kim, Yun-Tae Kim, Kyoung-Ryul Lee, Young-Jin Kim

**Affiliations:** ^1^Department of Bioanalysis, Seoul Medical Science Institute & Seoul Clinical Laboratories, Seoul 152-766, Republic of Korea; ^2^Department of Laboratory Medicine, Kwandong University College of Medicine, Gangneung 210-701, Republic of Korea; ^3^Department of Biological Engineering, College of Engineering, Konkuk University, Seoul 143-701, Republic of Korea

## Abstract

A fast, sensitive, and selective liquid chromatography-tandem mass spectrometry (LC-MS/MS) method was validated and then the levels of cortisol and cortisone from sera of healthy adults were determined by the LC-MS/MS method. One hundred ***μ***L of serum sample was directly extracted by adding 2 mL ethyl acetate, followed by chromatographic separation on a C18 column with a mobile phase consisting of 5 mM ammonium acetate and methanol (25 : 75, v/v). The precision, accuracy, and average recovery of the method were 1.5–5.3%, 95.4–102.5%, and 96.4% for cortisol, and 1.9–6.0%, 89.2–98.8%, and 79.9% for cortisone, respectively. The method was linear from 1.0 to 500.0 ng/mL (r^2^ = 0.999) for cortisol and 2.5 to 100.0 ng/mL (r^2^ = 0.998) for cortisone. The limits of detection (LOD) and quantification (LOQ) were 0.2 and 1.0 ng/mL for cortisol, and 1.0 and 2.5 ng/mL for cortisone, respectively. The average cortisol concentration (133.9 ± 63.7 ng/mL) of samples collected between 9:00 and 11:00 a.m. was higher approximately 4.4 times than that of cortisone (30.5 ± 10.7 ng/mL) (P < 0.0001). The average cortisone/cortisol ratio was 0.225. Therefore, the LC-MS/MS method may be useful for the diagnosis of some adrenal diseases and the assessment of 11***β***-hydroxysteroid dehydrogenase (11***β***-HSD) activity in clinical laboratories.

## 1. Introduction

Glucocorticoids are a class of steroid hormones that bind to the glucocorticoid receptor and contribute to the hypothalamic-adrenal-pituitary feedback system. They are part of the feedback mechanism in the immune system that turns immune activity down and therefore used to treat diseases caused by an overactive immune system, such as sepsis [1], allergies [2], autoimmune diseases, and asthma [3].

11*β*-Hydroxysteroid dehydrogenase (11*β*-HSD) is an enzyme that catalyzes the interconversion of physiologically active 11*β*-hydroxyl glucocorticoid, cortisol, and inactive 11-keto glucocorticoid, cortisone. Endogenous cortisol is reversibly converted to cortisone by 11*β*-HSD type 1 [4] whereas 11*β*-HSD type 2 predominantly catalyzes the conversion of cortisol to cortisone mostly in mineralocorticoid target tissues [5–8], and deficiency of this enzyme causes the syndrome of apparent mineralocorticoid excess (AME) [9, 10]. Cortisol is the most important glucocorticoid showing clinical immunologic, cardiovascular, homeostatic, and some metabolic functions [11–13]. Although cortisone is a more prevalent steroid in fetal tissues than cortisol, the level of cortisone decreases immediately after birth [14].

Cortisol and cortisone are measured directly from biological samples using immunoassays including radioimmunoassay (RIA), enzyme-linked immunosorbent assay (ELISA), and chemiluminescent immunoassay (CLIA) [15]. Among them, CLIA has become the most extensive method due to advantages such as automation, high throughput, and ease of use. However, it suffers from serious disadvantages such as sample matrix effects and lack of specificity resulting from cross-reactivity with structurally related endogenous steroids, lipids, or metabolites [16–18]. Therefore, a highly sensitive and specific analytical tool is needed to determine the concentrations of the two very similar molecules such as cortisol and cortisone in serum. In this respect, LC-MS/MS is becoming one of the most specific techniques available in clinical laboratories. LC-MS/MS also provides a robust platform with sufficient sensitivity and specificity for measuring steroid hormones simultaneously [17, 19].

Reference values of cortisol or cortisone vary between clinical laboratories due to the use of different analytical methods or in-house methods. Thus, validated assays are needed to measure glucocorticoid hormones accurately in samples originated from human. Although the levels of cortisol and cortisone in serum collected from healthy Japanese subjects between 9:00 and 11:00 a.m. were reported using high-performance liquid chromatography (HPLC) method [20], no study reported those in serum from Korean adults at the same time using LC-MS/MS method. At present, almost every clinical laboratory in Korea uses immunoassay-based methods to measure cortisol or cortisone in blood.

In this study, the assay method was validated following the recommendations outlined by the Food and Drug Administration (FDA) of the United States [21]. Evaluation of the method's performance included linearity, sensitivity, precision, accuracy, recovery, and interference. The aim of this study is to evaluate the 11*β*-HSD activity by measuring the concentrations of the two steroids in serum collected from Korean healthy volunteers between 9:00 and 11:00 a.m.

## 2. Material and Methods

### 2.1. Materials

Standards of cortisol (98%) and cortisone (98%) were purchased from Sigma Chemical Co. (St. Louis, MO, USA). Deuterated cortisol (cortisol-9, 11, 12, 12-*d*_4_, 98%) as an internal standard (IS) was purchased from Cambridge Isotope Laboratories, Inc. (MA, USA). Analytical-grade ammonium acetate (≥98%) and ethyl acetate (99.8%) and activated charcoal were purchased from Sigma. Gibco fetal bovine serum (FBS) was purchased from Life Technologies (CA, USA). HPLC-grade methanol and water were purchased from Fisher Scientific Korea Ltd. (Seoul, Republic of Korea). All solvents were filtered through Advantec membranes with 0.45 *μ*m pore size (Toyo Roshi Kaisha, Ltd., Tokyo, Japan).

### 2.2. Sample Collection

Forty-eight healthy adult volunteers (male = 6, female = 42, average age = 38) who had not received any hormone supplementation were recruited. All serum samples were collected at 9:00–11:00 a.m. using SST tubes (BD Inc., NJ, USA) and stored at −70°C until assay. Although most of the samples were collected from women, all the samples were studied as recruited, due to no meaningful difference between male and female in the concentrations of cortisol and cortisone [22]. Informed consent was obtained from all study participants, and the study protocols were approved by the Institutional Review Board of Seoul Medical Science Institute.

### 2.3. Standards and Sample Preparation

Stock solutions of cortisol, cortisone, and cortisol-*d*_4_ were prepared in methanol at a concentration of 100 *μ*g/mL. Working standards were prepared from the stock standards at a concentration of 1 *μ*g/mL. IS was prepared at a concentration of 0.1 mg/mL in methanol. Finally, the calibration standards were prepared in charcoal-stripped 5% FBS at concentrations of 1.0, 5.0, 10.0, 50.0, 100.0, and 500.0 ng/mL for cortisol and 2.5, 5.0, 10.0, 25.0, 50.0, and 100.0 ng/mL for cortisone. All the standards were stored at −20°C until assay. Sample preparation was a modification of two published procedures [23, 24]. Briefly, an aliquot of serum (100 *μ*L) was transferred into a glass tube and mixed with 20 *μ*L of working IS. For extraction, 2 mL of ethyl acetate was added into the tube. The tube was vortexed gently on a vortex mixer for 30 s and centrifuged at 3,000 rpm for 5 min. The upper layer was removed and then the lower organic layer was evaporated to dryness under nitrogen gas. No solid-phase extraction was done. The dried extract was reconstituted with 300 *μ*L of methanol, which was transferred to screw-capped injection vial.

### 2.4. LC-MS/MS Characteristics

HPLC was performed using an Agilent 1200 series (Palo Alto, CA, USA) equipped with an autoinjector and an autosampler. Separations of the steroids were performed on a Capcell Pak MG-II C18 column (3.0 mm,* i.d*., × 50 mm,* l*. 3 *μ*m particle size) (Shiseido, Tokyo, Japan). The injection volume was 5 *μ*L and the oven temperature was 25°C. The mobile phase consisted of 5 mM ammonium acetate and methanol (25 : 75, v/v) and was delivered at a flow rate of 0.25 mL/min. Mass spectral detection of positive ions in multiple-reaction monitoring (MRM) mode was performed using an API 4000 triple-quadrupole mass spectrometer (Applied Biosystems/MDS SCIEX, CA, USA) equipped with a Turbo V source and a TurboIonSpray probe. The following* m/z* MRM transitions were selected: 363.2 → 121.2 for cortisol, 361.2 → 163.2 for cortisone, and 367.1 → 121.1 for cortisol-*d*_4_. The declustering potential (DP), entrance potential (EP), collision energy (CE), and collision cell exit potential (CXP) were optimized at 79 V, 10 V, 33 V, and 6 V for cortisol, 111 V, 10 V, 33 V, and 30 V for cortisone, and 79 V, 10 V, 33 V, and 6 V for cortisol-*d*_4_, respectively. Ionspray voltage (IS) and temperature were 5500 V and 500°C, respectively. Collision gas (CAD), curtain gas (CUR), and ion source gases 1 (GS1) and 2 (GS2) were 6, 20, 60, and 45 psi, respectively. Peak areas of each analyte and the corresponding IS were obtained using Analyst 1.5 data processing software (Applied Biosystems/MDS SCIEX, CA, USA).

### 2.5. Method Validation

For the validation of the method's performance, linearity, LOD, LOQ, accuracy, precision, recovery, and interference were evaluated. Method validation was performed following the guideline outlined by the FDA [21].

#### 2.5.1. Linearity and Sensitivity

The linearity was evaluated by analyzing the regression coefficients of extracted cortisol and cortisone standard at 1.0, 5.0, 10.0, 50.0, 100.0, and 500.0 ng/mL and at 2.5, 5.0, 10.0, 20.0, 50.0, and 100 ng/mL, respectively. Each standard was analyzed in five different runs on five days. Serial dilution of a 5.0 ng/mL sample of cortisol and cortisone using charcoal-stripped 5% FBS was used to prepare the lowest concentration and to evaluate the LOD and LOQ. These samples were analyzed in ten replicates per run. The LOD and LOQ were determined as the lowest concentrations at which the analyte peaks were present at the expected retention times and the signal-to-noise (S/N) ratios >3 and >10, respectively. The limit of quantitation (LOQ) was determined as the lowest concentration for which accuracy was within ±20% and imprecision was within ±15%.

#### 2.5.2. Accuracy and Precision

Accuracy and precision are defined as the closeness between the concentrations of the analytes in a standard solution or in a spiked sample and the true concentrations and as the reproducibility of the signals observed by different analysis of an aliquot containing the analytes using a standard solution or spiked sample, respectively [25]. The accuracy and precision were determined from QC samples at four different concentrations of the two steroids (1.0, 5.0, 50.0, and 500.0 ng/mL for cortisol and 2.5, 5.0, 25.0, and 100.0 ng/mL for cortisone) including the LOQ concentrations. The QC samples were prepared from charcoal-stripped 5% FBS spiked with the four different amounts of cortisol and cortisone using stock solutions that were independent of those used to prepare the calibrators. The intra-assay accuracy and precision were evaluated by analyzing the three QC samples 5 times on 1 day. The interassay accuracy and precision were evaluated by analyzing the samples over five different days.

#### 2.5.3. Recovery

Recovery is defined as the closeness between the concentration observed by applying the present assay method to a spiked sample and the true concentration spiked to the sample matrix [25]. Recovery was evaluated using serum samples with low concentrations of the two steroids, spiked with the analyte standards at 5.0, 50.0, and 500.0 ng/mL for cortisol and 5.0, 25.0, and 100.0 ng/mL for cortisone.

#### 2.5.4. Interference

Interference studies were performed by spiking a pooled serum with 10 ng/mL of the following compounds: corticosterone, 11-deoxycortisol, progesterone, testosterone, 17-hydroxyprogesterone, dihydrotestosterone, aldosterone, and dehydroepiandrosterone. Interfering peaks at the cortisol and cortisone channels were identified.

### 2.6. Statistical Analysis

Data processing and graphic presentation were carried out with MS Office Excel 2007 (Microsoft Inc., Seattle, WA, USA). The concentrations of serum cortisol and cortisone were assessed statistically using the Student *t*-test. Statistical analysis was performed using GraphPad software (QuickCalcs, La Jolla, CA, USA). *P* < 0.05 was considered statistically significant.

## 3. Results and Discussion

### 3.1. LC-MS/MS Analysis

**Figure 1:**
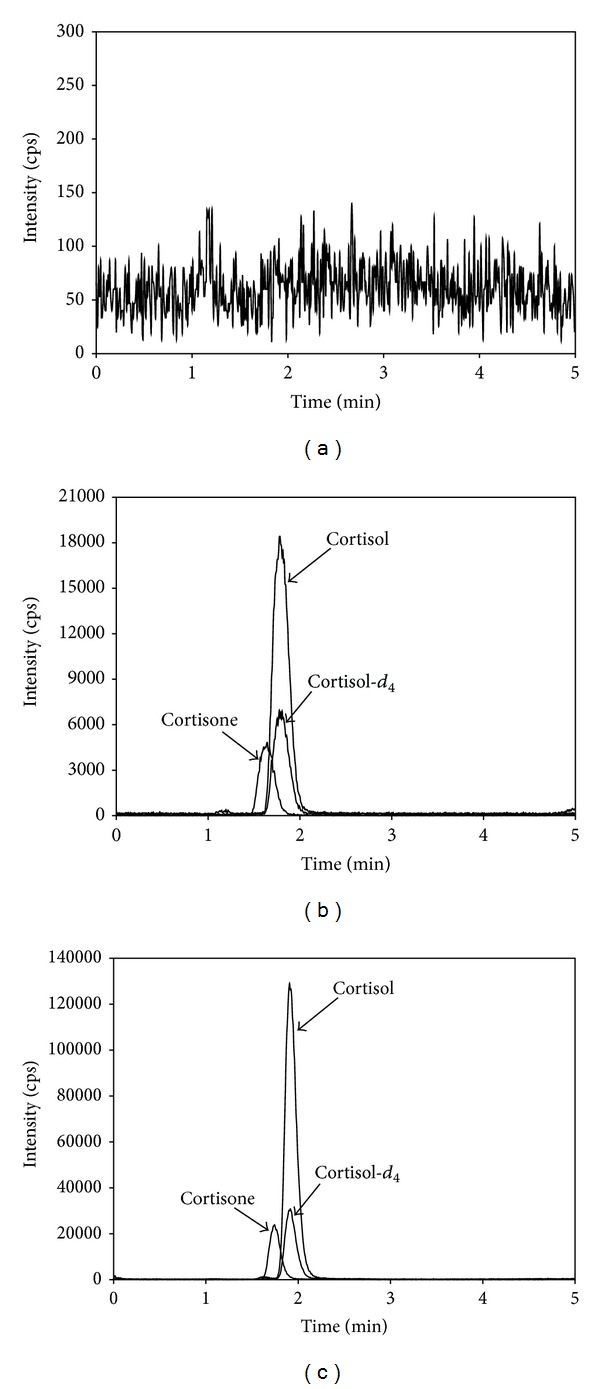
MRM chromatograms obtained by the present assay method. (a) Charcoal-stripped 5% FBS (blank). (b) A blank sample spiked with the standards at the concentrations of 10 ng/mL of cortisol and 10 ng/mL of cortisone and with 20 *μ*L of working IS. (c) A healthy subject serum with the concentrations of 25 ng/mL of cortisol and 50 ng/mL of cortisone.

It is needed to confirm that there are no interferences near the retention times of the analytes from a matrix as complex as serum [26, 27]. Therefore, the blank, the blank spiked with the standards (10 ng/mL for cortisol and 10 ng/mL for cortisone), and a serum sample from a healthy subject were analyzed. No peaks were observed in the blank sample (Figure 1(a)). In the MRM chromatogram of the blank spiked with the standards, cortisol, cortisone, and IS were fully separated within 3 min (Figure 1(b)). The retention times in the MRM chromatogram were 1.9 min for cortisol, 1.75 min for cortisone, and 1.91 min for cortisol-*d*_4_. The MRM chromatogram of a serum sample from a healthy subject showed that physiological components in the serum did not interfere with the identification and quantification of the analytes (Figure 1(c)). These results also indicate that the rapid analytical time and relatively small sample volume should facilitate high throughput measurement of both cortisol and cortisone simultaneously in human serum.

### 3.2. Method Performance

A simultaneous quantitative assay method for cortisol and cortisone in serum was validated. The calibration curves were linear in the range of 1.0–500.0 ng/mL for cortisol (*y* = (0.03431 ± 0.00471)*x* + 0.00783) and 2.5–100.0 ng/mL for cortisone (*y* = (0.02539 ± 0.00099)*x* − 0.00784). The weighed (1/*X*) least-squares determination coefficients were greater than 0.999 for cortisol and 0.998 for cortisone, indicating very good linearity.

The LOQ was found to be 1.0 ng/mL (2.75 nmol/L) for cortisol and 2.5 ng/mL (6.87 nmol/L) for cortisone (Table 1), indicating the values were within a biologically relevant range [23]. No ion suppression was observed at the retention times of the analytes.

The precisions (% coefficient of variations, CVs) and the accuracies of the LC-MS/MS method were determined by analyzing QC samples at four different concentrations for cortisol and cortisone (Table 1). Intra-assay (*n* = 5) CVs ranged from 2.7 to 4.6 for cortisol and 3.6 to 6.0 for cortisone, while accuracies (% bias) ranged from 95.4 to 102.5 for cortisol and 92.0 to 98.8 for cortisone. Interassay (*n* = 5) CVs ranged from 1.5 to 4.5 for cortisol and 1.9 to 5.8 for cortisone, while accuracies ranged from 97.3 to 100.4 for cortisol and 89.2 to 96.0 for cortisone. These results indicate that the CVs and the accuracies were within internationally accepted criteria [21]. The recoveries of cortisol were 99.0, 96.1, and 94.0% in serum samples spiked with the standards at 5.0, 50.0, and 500.0 ng/mL, respectively. The recoveries of cortisone were also 77.1, 81.6, and 81.2% in serum samples spiked with the standards at 5.0, 25.0, and 100.0 ng/mL, respectively (Table 1).

### 3.3. Quantitative Analysis of Sera from Subjects

The mean concentration of cortisol (133.9 ± 63.7 ng/mL) was about 4.4 times higher than that of cortisone (30.5 ± 10.7 ng/mL) at 9:00–11:00 a.m. (*P* < 0.0001) (Table 2). The mean concentrations of cortisol and cortisone were 132.9 and 27.8 ng/mL for male and 134.0 and 31.0 ng/mL for female, respectively. These results are similar to the established reference intervals for cortisol and cortisone in healthy American subjects by LC-MS/MS assay [23, 24] or in healthy Japanese subjects by HPLC assay [20, 28]. In our study, no significant differences between male and female's cortisol or cortisone levels were found, indicating that the levels of cortisol or cortisone in serum are regardless of gender [22].

**Figure 2:**
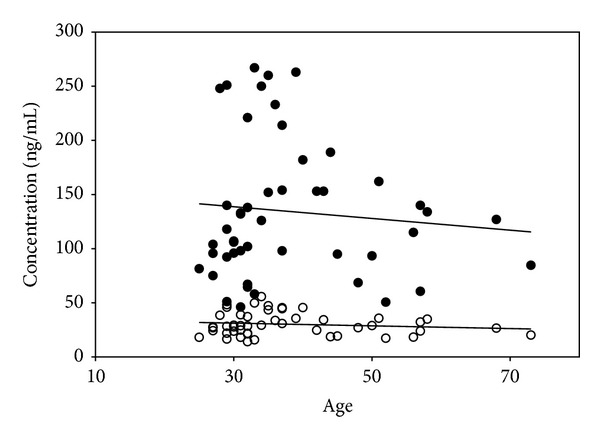
The levels of serum cortisol (●) and cortisone (○) with age in subjects.

Furthermore, the concentrations of cortisol and cortisone were not notably changed with increasing age (Figure 2). In a previous report, although healthy Japanese adults had higher levels of cortisol and cortisone than healthy children from 1 to 19 years old, the difference was not statistically notable [28].

**Figure 3:**
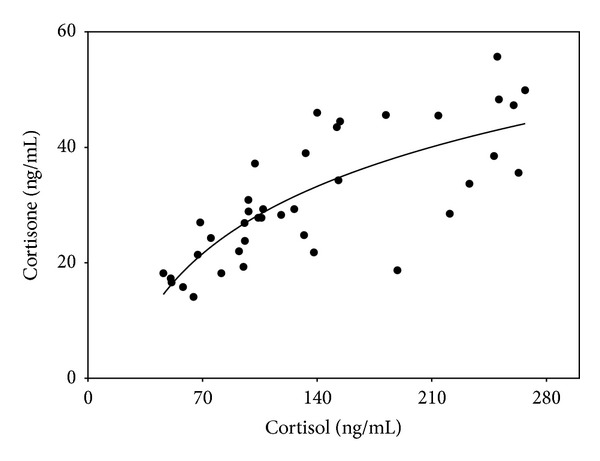
Comparison of the concentration levels between serum cortisol and cortisone collected between 9:00 and 11:00 a.m.

The concentrations of serum cortisol were compared with those of serum cortisone (Figure 3). The fitted curve showed a tendency towards a plateau at higher concentration levels, which might indicate the saturation of the 11*β*-HSD type 2 at high concentrations of the substrate [29]. This result shows a status of cortisol-cortisone shuttle in serum and also gives an evidence for the activity of 11*β*-HSD type 2 that catalyzes the irreversible conversion of active cortisol into inactive cortisone [30].

No significant differences were found between males and females in the cortisone/cortisol ratios. The average ratio of males and females in this study was 0.225 (Table 2). This result showed similar pattern to the ratios previously measured in sera from 69 healthy Japanese subjects [20]. It is well known that the cortisone/cortisol ratio is decreased in hyperadrenalism and under physiological stress but is increased in hypoadrenalism. Therefore, the cortisone/cortisol ratio can give useful information in evaluating the adrenal function of patients with various diseases [20, 22, 24, 28].

## 4. Conclusions

In this study, the levels of cortisol and cortisone in the sera from healthy Korean subjects were simultaneously measured using a validated LC-MS/MS method after a simple liquid-liquid extraction. To the best of our knowledge, this work is the first report on simultaneous measurement of cortisol and cortisone in the sera from healthy Korean subjects by LC-MS/MS. The present LC-MS/MS method is rapid, sensitive, specific, and robust for the simultaneous measurements of cortisol and cortisone in serum. The method also may be cost-effective compared with the previous reports using any solid-phase extraction cartridge [31] or online extraction equipment [32]. The level of serum cortisone was lower about 4 times than that of cortisol and the average cortisone/cortisol ratio was 0.225. Also, the method may provide valuable information about 11*β*-HSD activity in the study of cortisol-cortisone shuttle. Therefore, the LC-MS/MS method could be an alternative method to conventional enzyme immunoassays for the diagnosis of several adrenal dysfunctions in routine clinical laboratories.

## Figures and Tables

**Table 1:** Method validation results of the LC-MS/MS assay.

	Concentration (ng/mL)	Intra-assay (*n* = 5)		Interassay (*n* = 5)		Recovery (%)	LOQ (ng/mL)
		Accuracy (%)	CV (%)	Accuracy (%)	CV (%)		
Cortisol	1.0	95.4	4.6	97.3	4.5		1.0
	5.0	99.2	2.9	100.4	2.1	99.0	
	50.0	102.5	5.3	100.4	1.6	96.1	
	500.0	96.4	2.7	97.3	1.5	94.0	

Cortisone	2.5	98.8	6.0	92.8	5.8		2.5
	5.0	94.0	4.5	90.1	2.9	77.1	
	25.0	92.0	5.6	89.2	1.9	81.6	
	100.0	97.3	3.6	96.0	2.3	81.2	

**Table 2:** Mean concentrations (ng/mL) of cortisol and cortisone in serum collected between 9:00 and 11:00 a.m.

	Mean age	Cortisol (range)	Cortisone (range)	Cortisone/cortisol ratios	*P* value
Male (*n* = 6)	52.3	132.9 (93.4–162.0)	27.8 (18.3–35.8)	0.209	
Female (*n* = 43)	35.2	134.0 (46.1–267.0)	31.0 (14.1–55.7)	0.231	
Total (*n* = 49)	37.3	133.9	30.5	0.225	*P* < 0.0001
